# Fracture of Skull with Complications

**Published:** 1907-02

**Authors:** A. M. Rooker

**Affiliations:** Buffalo. N. Y.; House Surgeon of the German Hospital


### CLINICAL REPORT.

### Fracture of Skull with Complications.

Reported by A. M. ROOKER, M. D., Buffalo, N. Y.
House Surgeon of the German Hospital.

J. H., male; aged 37 years, while intoxicated fell at his work, injured his head and being dull and stupid as a result was brought to the hospital. Physical examination showed an abrasion on the scalp over right eye, the skin being scraped and the scalp ecchymosed. Around his right ear there was marked edema; no blood from ears, nose or mouth, nor were there any eye symptoms indicative of fracture. Temperature, 97.8; pulse 94; respiration 22. The urine, 1047 sp. gr., contained a large amount of sugar, otherwise normal.

Soon after entering the hospital he developed a well marked case of delirium tremens, running a typical course. In two days a hematoma developed under the scalp of the vertex; this was incised, the clot turned out, the bleeding vessel ligated and the wound sutured. While the scalp was open an examination was made for fracture but none detected. The unconsciousness persisted and deepened, the temperature and pulse gradually increasing until, on the seventh day when with a temperture of 105.5, pulse 144, and respiration 72, he died.

The postmortem showed an extradural bloodclot covering the entire vertex, being fully five inches in diameter and over an inch in thickness, the bleeding having taken place from both meningeal arteries which were torn by a fracture extending across the entire vertex through both internal and external plates, following the coronal suture for an inch and one half on either side of the median line, (for this reason it was not detected when scalp was incised for hematoma) and extended on either side down to, but not into the petrous portion of the temporal bone, the base being uninjured.

The case is especially interesting because of the occurrence of so extensive a clot with no visible symptoms of pressure except unconsciousness, and this could be readily explained by the delirium tremens. Then, too, the glycosuria adds another possibility; might not death have been due to diabetic coma? No history, however, was obtainable and the urine could not be watched because of incontinence with restlessness and mania.

On the death certificate, fracture of the skull was given as the cause of death. Might not delirium tremens or diabetes have been contributing causes?

